# Early urban impact on Mediterranean coastal environments

**DOI:** 10.1038/srep03540

**Published:** 2013-12-18

**Authors:** David Kaniewski, Elise Van Campo, Christophe Morhange, Joël Guiot, Dov Zviely, Idan Shaked, Thierry Otto, Michal Artzy

**Affiliations:** 1 Université Paul Sabatier-Toulouse 3, EcoLab (Laboratoire d'Ecologie Fonctionnelle et Environnement), Bâtiment 4R1, 118 Route de Narbonne, 31062 Toulouse cedex 9, France; 2 CNRS, EcoLab (Laboratoire d'Ecologie Fonctionnelle et Environnement), 31062 Toulouse cedex 9, France; 3 Institut Universitaire de France, 103 boulevard Saint Michel, 75005 Paris, France; 4 Aix-Marseille University, CNRS, UM 34, Europôle de l'Arbois BP80, F-13545 Aix-en-Provence, France; 5 Leon Recanati Institute for Maritime Studies, University of Haifa, Mount Carmel, Haifa 3190501, Israel; 6 Hatter Laboratory, University of Haifa, Mount Carmel, Haifa 3190501, Israel

## Abstract

A common belief is that, unlike today, ancient urban areas developed in a sustainable way within the environmental limits of local natural resources and the ecosystem's capacity to respond. This long-held paradigm is based on a weak knowledge of the processes underpinning the emergence of urban life and the rise of an urban-adapted environment in and beyond city boundaries. Here, we report a 6000-year record of environmental changes around the port city of Akko (Acre), Israel, to analyse ecological processes and patterns stemming from the emergence and growth of urban life. We show that early urban development deeply transformed pre-existing ecosystems, swiftly leading to an urban environment already governed by its own ecological rules and this, since the emergence of the cities.

The question of whether or not the emergence of cities^1^,^2^ ~6000 years ago and the subsequent early urban growth in Western Asia^3^,^4^,^5^,^6^,^7^ deeply impacted natural environments and pushed them towards irreversible changes, has gained new interest because of the recent reassessment of ecological interactions caused by and taking place in urban environments^8^,^9^,^10^,^11^,^12^,^13^. Urban developments have been emergent phenomena of local-scale, evolving as the outcome of dynamic interactions among biophysical, human and socio-economic forces in which each component contributes to but does not control the form and behaviour of the whole^14^,^15^. Since the emergence of cities, the growing urban context has gradually shaped the socio-ecological interactions that have been both drivers and responders to long-term environmental changes^11^,^14^. Urbanization is one of the dominant demographic trends of our time, and more than 50% of humans will be concentrated in cities in 30 years time, as a result of increasing human population and migration from rural to urban areas^11^,^13^. Urban growth involves one of the most extreme forms of ecological stress and land alteration^10^. Humanity has long experienced a shift towards urban living^2^,^3^,^4^,^5^,^6^, but it is still equivocal whether the process of economic development was based upon the realities of the available ecological capital, or if such changes acted like a trigger to a profound environmental degradation. As new urban restoration targets focus on the temporal and spatial dimensions of the ecological history^16^, a thorough knowledge of the early interactions between natural communities and human activities in and around an emerging city, may help recovering the original patterning.

Here, the emergence of an urban environment as a unique setting was analyzed through the investigation of patterns of pressure and selection on ecosystems on the coastal strip of Israel, the ancient and present economic heart^17^ of a country where 91.9% of the population live in urban areas^18^. With high density urban development and relatively scarce land resources, the coast and seashore are vital open-spaces. The port city of Akko (Acre), a UNESCO world heritage site, is one of the oldest continuously inhabited places and a strategic link to the Levant (Supplementary Fig. S1a–b), leading eastward to the Jordan Valley and further to Transjordan^19^,^20^,^21^,^22^. Except for short periods, Akko was an important Eastern Mediterranean administrative and trading center from ~4000 calendar years before the present (BP) to the Ottoman period. The ecological impact of the spurt of urbanization on and beyond the most ancient site, located on a tell at a short distance from modern Akko, was reconstructed from a continuous and well-dated (Table 1) 6000-year record of ecosystem dynamics (Fig. 1a).

## Results

**Figure 1:**
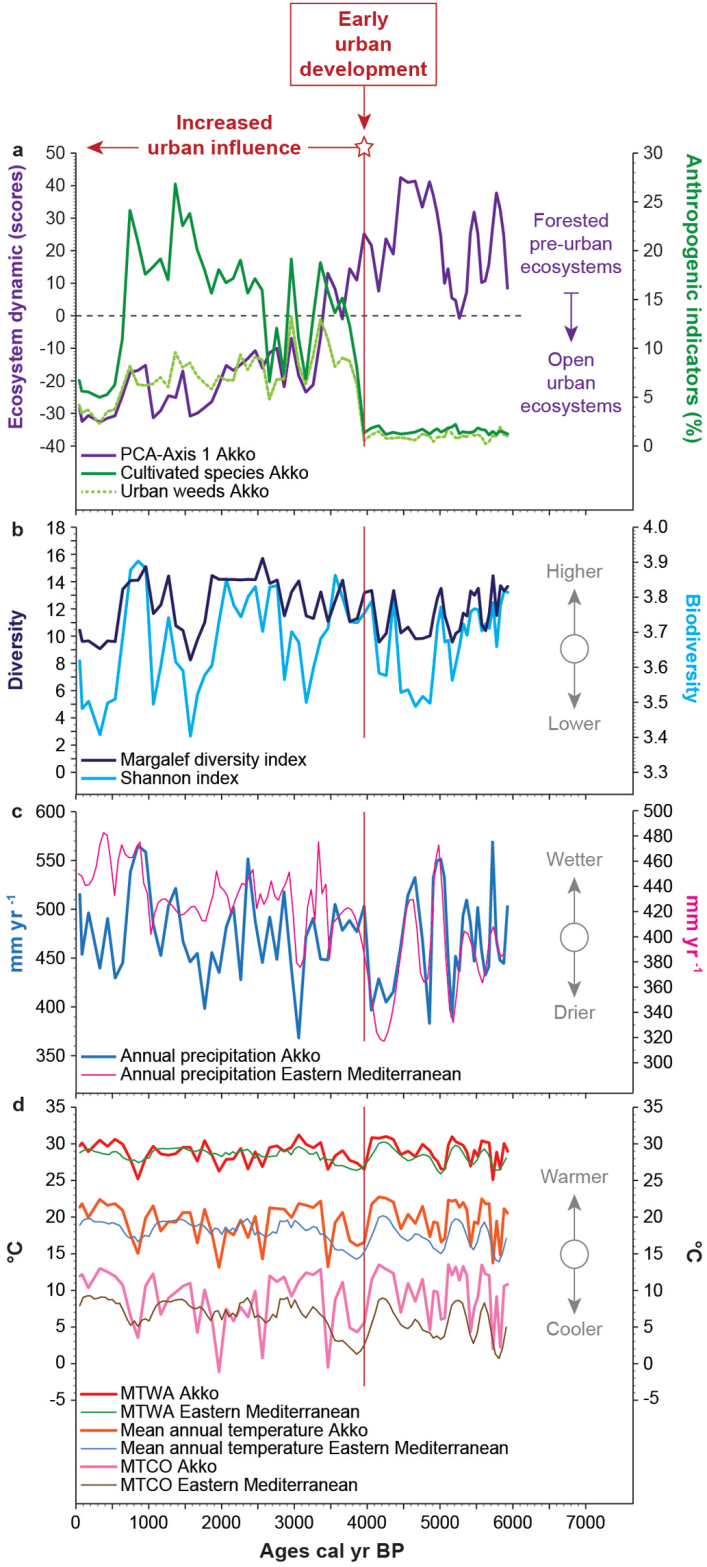
A 6000-year environmental reconstruction from Akko. (a) Pre-urban (positive scores) *versus* urban-adapted ecosystems (negative scores), with anthropogenic indicators (cultivated species and urban weeds) plotted on a linear age-scale. (b) Diversity indexes (Margalef and Shannon). (c) Annual precipitations reconstructed for Akko and the Eastern Mediterranean. (d) Mean annual temperatures, mean temperature of the coldest month (MTCO) and mean temperature of the warmest month (MTWA) reconstructed for Akko and the Eastern Mediterranean. All the 90%-confidence intervals are displayed in Supplementary Figs. S4–5.

**Figure 2:**
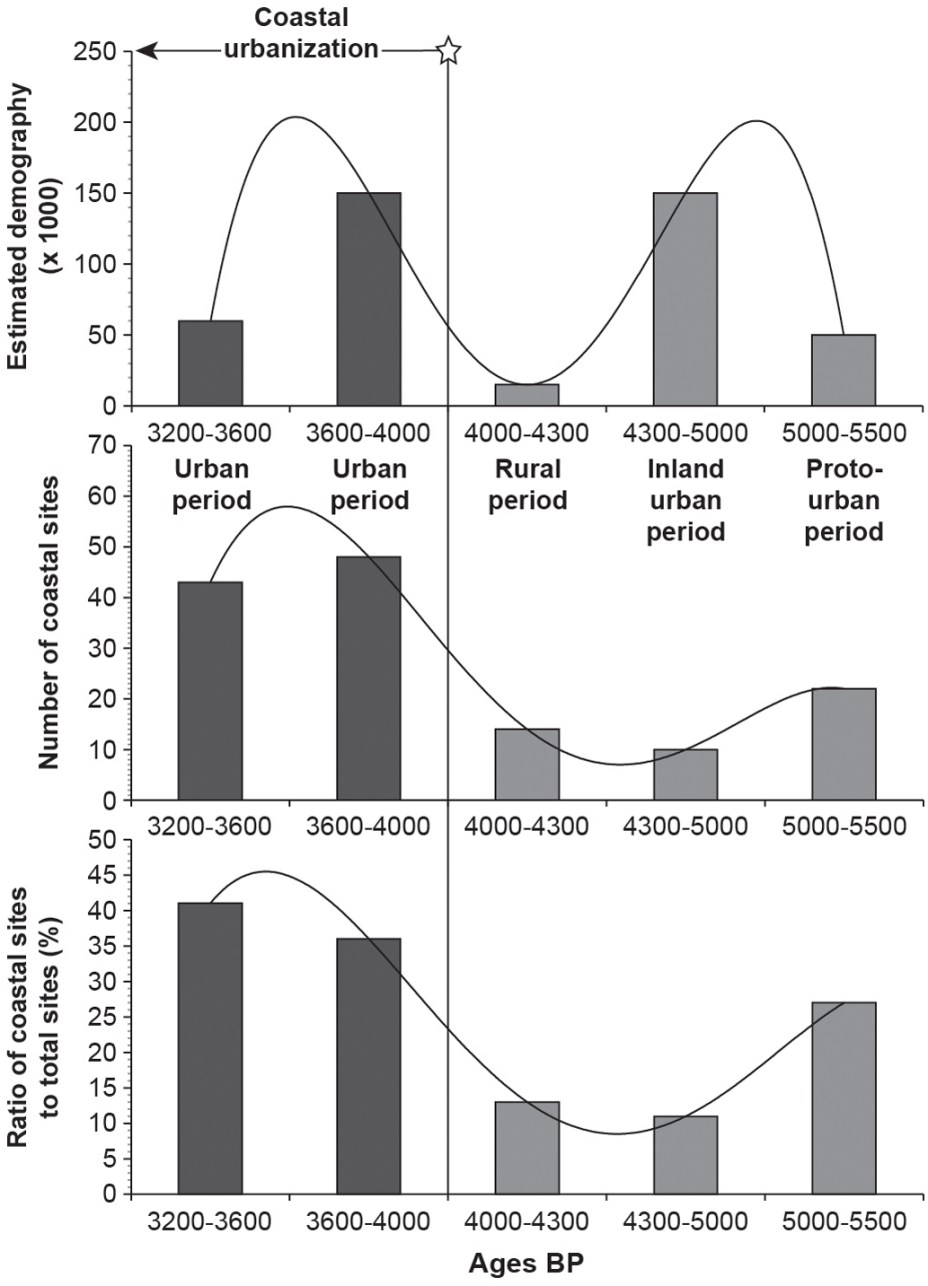
Estimated demography for the 5500–3200 BP period in Israel with the number of coastal sites. The ratio coastal *versus* inland sites gave an historical overview of the urbanization of the littoral. The polynomial curves give global trends for the period 5500–3200 BP. Data were compiled from different sites in Israel^45^.

An outstanding feature of the recorded urban environmental history of Akko is that the area rapidly shifted from a densely forested landscape to a shrub-steppe (Fig. 1a–b) between ~4000 and 3300 calibrated years before the present (cal yr BP). The regional climatic trends (Fig. 1c–d) do not support climate as a primary cause for this shift, since no decreasing precipitation (Fig. 1c), consistent with a forest-steppe shift, is observed for this period. Higher amounts of precipitation at Akko since ~4000 cal yr BP are also recorded in the Eastern Mediterranean by low δ^18^O values on Ashdod Coast^23^,^24^ and Soreq Cave^25^. Higher precipitation in the Dead Sea^26^ and in the Sea of Galilee^27^, as well as increased Nile floods^28^ and a wet phase in coastal Syria^29^ all suggest that the 4000–3300 cal yr BP period is inconsistent with climate pressure as a forcing factor behind the forest-steppe shift. Only a background of changes in coastal morphology due to relative sea level changes^30^,^31^ (Supplementary Fig. S1a), which also controlled the evolution of the coastal ecosystems in and around Akko (Fig. 1a), are recorded between 4000 and 3300 cal yr BP. Although the coastal mobility of the Haifa Bay was one of the forcing factors that impacted upon ecosystem dynamics since 8500 cal yr BP^32^, the final stage of ecological erosion at the site, a permanent open shrub-steppe (Supplementary Fig. S2), was only reached after the emergence of the city (Fig. 1a). The first interventions, substitutions and transformations of the pristine Mediterranean forest promptly followed the occurrence of the earliest urban structures, dated from the Middle Bronze Age IIA culture (~4000 BP). The local urbanization occurred at the termination of the 4200 cal yr BP drought event^33^,^34^,^35^ (Fig. 1c), paralleling the dense occupation phase that appeared along the southern Levantine coastal area (Fig. 2). The city rapidly developed with ramparts, buildings and industrial areas^20^,^21^,^22^ (Supplementary Fig. S1b). The anchorage, in connection with the Na'aman River fluvial system, was the focus of the economy and trade, and the main driver behind urban population growth. The spatial concentration of agricultural, industrial and commercial activities led to increased demands on local ecosystems (Fig. 1a), and to an encroachment on and a loss of natural biotopes in and around the tell. Fragmented proto-urban ecosystems only persisted as small patches within a matrix of urban and agricultural expansion (Supplementary Fig. S3a–b), or even disappeared. This human-induced ecological imbalance has prevailed throughout the last ~4000 years, as the coastal vegetation became dominated by a dry and urban-adapted woodland (Supplementary Fig. S3c) in association with a shrub-steppe, which became prevalent during two periods, 1670 to 1060 cal yr BP and 430 to 100 cal yr BP.

During the first millennium of human occupation, a sharp decrease in agricultural productivity at 3250–3200 cal yr BP (Fig. 1a) is associated with a weakening of the economy and reduction in habitation at Akko. The 3200 cal yr BP drought event^36^,^37^ had affected most of the Eastern Mediterranean and adjacent regions^26^,^27^,^28^,^29^. Even if the patterns of sprawl were primarily controlled by humans since their settlement at Akko, at the end of the Late Bronze Age (~3200 BP), the cumulative influence of reduced precipitation (Fig. 1c), coastal progradation^38^ due to slower relative sea-level rise^39^, and the continuous Nile-derived sand deposition in Haifa Bay area with the silting up of the Na'aman River mouth^38^, generated a drier coastal area and the subsequent development of a dry steppe at ~3160 cal yr BP. These processes have constrained the rate of urban and economic development, stressing the limiting role of coastal changes (progradation, silting up of the river mouth) and water availability (surface *versus* groundwater; depending on functional pits) for urban growth. Although the 3200 cal yr BP event locally ended at ~2900–2800 cal yr BP, the re-emergence of extensive habitation pattern and trade networks only occurred after ~2650 BP, during the Phoenician/Persian and Early Hellenistic periods, and is clearly marked in the environment (Fig. 1a).

A second sharp decrease in agricultural productivity was recorded since ~650 cal yr BP, at the end of the Crusader period-onset of the Mamluk-Ottoman era. Following the fall of the Crusaders, the area was left untilled and under urbanized for a long time. Akko was replaced by Safed as the administrative city of the Mamluks. During the Ottoman period, Akko was described by pilgrims and merchants who visited it in the 16^th^ and 17^th^ centuries as a deserted ghost town, with some structures from the Crusader period still standing, some jutting out of the earth, and others buried. Regardless of the causes behind the decay of Akko since 650 BP, the lack of resilience of coastal ecosystems following the abandonment of the town may result from 3500 years of continuous human pressure, leading to an ecological erosion marked by non-regenerating stands of forested communities. The dry conditions of the Little Ice Age are only recorded after ~500 cal yr BP in the Levant^23^,^24^, during the Ottoman Empire^40^,^41^, and cannot be the main forcing agent behind the permanent open shrub-steppe recorded since the fall of the Crusaders.

## Discussion

Humans are a key component of ecosystem dynamics^14^,^42^ in and around the site of Akko through an urbanization process that began ~4000 cal yr BP (Fig. 2), with a background of climate changes and coastal mobility. By adding selection forces to these natural phenomena (climate and coastal mobility), early urbanization gradually changed the expression of ecological processes governing the coastal environmental dynamics before the settlement (Fig. 1a). Plant richness increased in the urban environment (Fig. 1b), due to the highly heterogeneous patchwork of habitats^8^, and the potential co-existence between natural and anthropogenic-favoured species. Whereas proto-urban coastal ecosystems were defined on the basis of variables such as soil, temperature, precipitation and dominant vegetation types (Fig. 1a–d), we show how the urban process (Fig. 2) swiftly generated or amplified a biotic imbalance which increased the biological diversity (Fig. 1b), but reduced the uniqueness and the resilience of the proto-urban ecosystems^43^.

Accelerated population growth since ~4000 BP^21^ (Fig. 2) and unsustainable development generated by socio-economic demands dramatically increased water needs^11^,^14^. Higher water uptake from watercourses and water tables, associated with the intentional or unintentional anthropogenic pressures on the fertile alluvial plains of the Na'aman River, the main source of freshwater at Akko, may further explain the expansion of an urban-adapted shrub-steppe. Increased water demand and changes in land use altered the ability of natural reservoirs to fully mitigate the impact of environmental changes. Two positive feedbacks may have reinforced this process. Firstly, the human-induced fall in free water or vegetated surfaces would have lowered the heat loss due to evaporative cooling, increasing local temperature. Secondly, an increase in solar absorption by urban buildings, having higher heat capacities than natural environments and a lower albedo, would also have raised local temperatures (urban heat island)^11^, even if this process was probably limited during the Bronze Age compared to Hellenistic-Roman and later periods. Environmental stress on ecosystems already undermined by human activity (Fig. 1a), produced a specific urban-based vegetation dynamic in the Akko area.

The detailed reconstruction of ecological changes along a rural-urban gradient at the harbour site of Akko provides a unique model to explore the effects of disturbances due to urban dwellers on native vegetation communities since the early rise of cities in the Near East. Land-use changes were likely driven by the first population movements that occupied Akko, but it is only after the foundation of the city that evolving socio-ecological agents, connected with changing human activities and patterns of population densities (Fig. 2) in and around the site, deeply altered the environmental dynamics and natural community structures. This questions the long-held belief of a “golden age” of sustainable early urban development. The same mechanisms that degrade or overexploit the ecosystems nowadays were already at work, even if technologies and agro-innovations were markedly different during the pre-industrial era. Accepting large urban concentrations might need to concede an intrinsic impossibility to produce locally sustainable development. The 4000-year urban history of Akko illustrates the growth of an urban-adapted environment governed by its own ecological rules, based on the loss of particular combinations of populations and species, the fragmentation of natural ecosystems and reduced water allocations. Impairment of ecosystem services and loss of biodiversity assets of potential economic significance was the price to pay for these first urban civilizations to ensure a socio-economic development and an expansion of their trade networks. Sustainability was, since the beginning, a utopian goal as landscapes have evolved continuously according to social and economic needs of a particular society at a given moment.

## Methods

### Core lithology and chronology

We used biological indicators (pollen grains, fern spores, micro-charcoal fragments, and dinoflagellate cysts) extracted from a continuous core (32°54′N, 35°05′E; +3 meter above sea level) drilled on the southern flank of Tel Akko (Supplementary Fig. S1a–b), close to the “old city” of Akko to reconstruct the ecological urban history of the southern Levant. This core was selected from a North-South transect between the foot of the tell and the Na'aman River. The sedimentary deposits (215 cm) mainly consist of silts and clays, with scarce sand inclusions. The chronology is based on five accelerator mass spectrometry (AMS) radiocarbon (^14^C) ages of short-lived terrestrial samples (Table 1). All conventional radiocarbon ages have been calibrated [one and two sigma (σ) calendar calibration] using Calib-Rev. 6.0.1^44^. Compaction corrected deposition rates have been computed between the intercepts of adjacent AMS ^14^C ages. The age of each sample was calculated by interpolation.

### Archaeological and historical data

Archaeological and historical data for urban development derive from 40 years of excavation at the ancient site of Tel Akko (since the work of M. Dothan) and in the “old city”, especially the crusader town (since the surveys of the British Mandate Authorities).

### Pollen analyses

A total of 70 samples were prepared for pollen analysis using the standard palynological procedure for clay samples. Pollen grains were counted at ×400 and ×1000 magnification using an Olympus microscope. Pollen frequencies (%) are based on the terrestrial pollen sum excluding local hygrophytes and spores of non-vascular cryptogams. Aquatic taxa frequencies were calculated by adding the local hygrophytes-hydrophytes to the terrestrial pollen sum.

### Numerical analyses

Biological data were analysed using Cluster analysis (CA), Kernel density-2D, Matrix, and Principal Components Analysis (PCA). The CA is a technique for hierarchical clustering, finding ranked groupings in multivariate data sets. Here, it is based on pollen-type time-series (presence/absence and abundance). The CA (Supplementary Fig. S2) was used to compute the lengths of branches of a tree, using branches as ecological distances between groups of taxa. CA was computed using *Paired group* as algorithm and *Spearman's Rho* (r value of the ranks) as similarity measure. The pollen-types from each cluster were summed to create nine pollen-derived ecosystems (Supplementary Table S1).

The ordination of pollen-derived ecosystems was tested using Kernel density-2D with *Gaussian* as a basis function and three units as *radius* (Supplementary Fig. S3a–b), and displayed as a two-dimensional plot of the data matrix (percentages; Supplementary Fig. S3c). The Kernel density estimation is a nonparametric technique for density estimation. It represents a generalisation of histogram density estimation with improved statistical properties.

PCA was then performed to test the ordination of samples by assessing major changes in the pollen-derived ecosystems^29^. The main variance is loaded by the PCA-Axis1 (Supplementary Table S2). Mediterranean open-forest, *Quercus calliprinos* woodland and shrub-steppe (natural *versus* urban-adapted) correspond to the main loadings in the PCA, explaining most of the variance for the PCA-Axis 1 ordination of the data, which accounts for +0.777 of total inertia. Natural ecosystems [Mediterranean open-forest (+0.84), wet meadow steppe (+0.13), *Quercus ithaburensis* forest (+0.06) and fen trees (+0.01)] are loaded in positive values, whereas negative values correspond to urban-adapted ecosystems [phrygana-batha (−0.02), dry steppe (−0.04), *Quercus calliprinos* woodland (−0.35) and shrub-steppe (−0.39)]. The PCA-Axis 1 scores have been plotted on a linear age-scale to determine the main changes in the Akko core (Fig. 1a).

### Climate reconstruction

The climatic data (Supplementary Table S3) used are monthly temperature, precipitation and cloud cover gridded at a step of 0.5° from 1901 to 2000, provided by the British Atmospheric Data Centre (CRU-TS-3-10)^46^. Pollen diagrams at five sites are considered, Tell Tweini^29^, Bereket^47^, Jableh^40^, Hala Sultan Tekke^37^, and Akko. The closest point of the climatic grid is assigned to each of the five pollen sites. We use a vegetation model (BIOME4)^48^ to estimate the net primary productivity of vegetation in equilibrium with the climate of each of the studied sites. Assuming that there is a relationship between these productivities and the pollen abundances, and that the corresponding residuals have a Gaussian distribution, we estimated the climate scenarios that provide the best fit relationships. This procedure, called model inversion, is solved by a Bayesian approach^49^. Given likelihood comparing model outputs to data and prior parameter models, Markov Chain Monte Carlo (MCMC) technique produces an ensemble of draws from the posterior distribution, from which estimates of the parameters and their associated uncertainties can be made^50^. The parameters are the 36 monthly climatic variables which are summarized into a smaller number of parameters to decrease the complexity of the problem. We used the first four principal components. A large number of iterations (>10,000) are required to analyse the posterior probability distributions (we assume that they are independent). For each pollen assemblage (aggregated into 13 groups compatible with the outputs of BIOME4), and at each iteration, the four parameters are transformed into a 36-size climatic vector which is introduced at the input of BIOME4 (together with the actual CO_2_ concentration, estimated from ice cores, and modern soil characteristics). A simulation of each vegetation assemblage is obtained and compared to the pollen data. Acceptable scenarios (according to MCMC rules)^51^ are kept to increment the posterior distributions of the climate parameters. Verification is done on 11 modern spectra from the southeast Mediterranean region with modern top core available (four from this study, seven from the European Pollen Database). We have calculated the root-mean-square error (RMSE) and the bias (Supplementary Table S4). It appears that the RMSE is high for the mean temperature of the coldest month and the annual temperature. This is due to a large bias, *i.e.* a strong underestimation of the winter temperatures, because several tree taxa cannot be unequivocally assigned to cool or warm conifer plant types^52^; then they are assigned to both. The reconstructed variables are corrected by subtracting the bias, and the corrected biases are more acceptable. They are inferior or equal to the 90% confidence intervals of the reconstructions. That means that, after correction for the respective biases, the modern climate reconstructed by pollen and inverse modelling is not significantly different from the modern climate. The reconstructions for the Holocene (Supplementary Tables S2, S5) are then corrected by the estimated modern climate (top core), and then added to the observed modern climate in Akko or Eastern Mediterranean (Fig. 1c–d, Supplementary Figs S4–5).

## Author Contributions

D.K., E.V.C., C.M. and M.A. had the idea for the research. D.K. and E.V.C. performed the research, undertook the data collection, and led the statistical analysis. J.G. led the climate reconstruction. D.K., E.V.C., C.M., J.G., D.Z., I.S., T.O. and M.A. contributed to the analysis and interpretation of the results. D.K. wrote the first draft of the paper and all authors contributed to writing the manuscript.

## Supplementary Material

Supplementary Information Supplementary Figures and Tables

## Figures and Tables

**Table 1:** Details of the radiocarbon age determinations for the Akko core. The radiocarbon ages are expressed in calibrated year BP at 68–95% of probability

Akko core			Calibrated dates BP		Intercepts
Code	Depth cm	^14^C yr BP	1σ-68%	2σ-95%	Cal yr BP
BETA-337808	80	190 ± 30	190-160	225-140	170
BETA-337809	101	1310 ± 30	1290-1255	1295-1220	1270
BETA-347581	133	2770 ± 30	2890-2840	2950-2790	2860
BETA-347582	173	4330 ± 30	4890-4850	4970-4840	4860
BETA-337810	215	5200 ± 30	5950-5920	6000-5910	5930
