# Supplementary material for: Early urban impact on Mediterranean coastal environments
Source: Sci Rep. 2013 Dec 18;3:3540. doi: 10.1038/srep03540 (PMC3866597; doi:10.1038/srep03540)
Supplement: Supplementary Information — Supplementary Figures and Tables [file srep03540-s1.pdf]

Supplementary Materials for

Early urban impact on Mediterranean coastal environments

David Kaniewski, Elise Van Campo, Christophe Morhange, Joël Guiot, Dov Zviely, Idan Shaked, Thierry Otto, Michal Artzy

This file includes:

5 figures

5 Tables

Supplementary Figures and Tables

***Supplementary Figure S1*│ Map of northwestern Israel and of the evolution of urbanization at Tel Akko and Akko since ~ 4000 BP.** The map was generated by D. Zviely with Adobe Illustrator CS5 (**a**) Details of the Haifa Bay[41] with the tells indicated as yellow circles. The palaeocoastline for the 4000-3000 cal yr BP period, termed Bronze Age coastline, is underlined in red. (**b**) Urban development at Akko since ~ 4000 BP with the building of a massive rampart during the Middle Bronze Age (picture M. Artzy), a public building from the Persian period (picture M. Artzy) where several ostraca written in Phoenician were found, and the "old city" in 1918. Akko is nowadays an expanding urban center.

***Supplementary Figure S2* │ Pollen-derived ecosystems established from the main taxa from the Akko record.** A total of 9 clusters were defined by the neighbour joining. The pollen-types of each cluster were summed to define 4 pre-urban and 5 urban-adapted pollen-derived ecosystems. The three main pollen-derived ecosystems in the coastal area for the last 6000 years are Mediterranean open-forest, *Quercus calliprinos* woodland and shrub-steppe.

***Supplementary Figure S3* │ Main transition towards an urban environment highlighted by dominant pollen-derived ecosystems.** The pre-urban Mediterranean open-forest is plotted against two urban-adapted ecosystems [(**a**) *Quercus calliprinos* woodland, (**b**) shrub-steppe]. The urban threshold, corresponding to the main shift in vegetation cover, is underlined by a white stripe. (**c**) Reconstructed ecosystems (matrix %) with the natural ecosystems on the left side and the urban-adapted on the right side.

***Supplementary Figure S4* │ Akko climate reconstruction.** Climate reconstruction with 90%-confidence interval of the annual precipitation, mean annual temperature, mean temperature of the coldest month (MTCO) and mean temperature of the warmest month (MTWA).

***Supplementary Figure S5* │ Eastern Mediterranean climate reconstruction.** Climate reconstruction with 90%-confidence interval of the annual precipitation, mean annual temperature, mean temperature of the coldest month (MTCO) and mean temperature of the warmest month (MTWA). The reconstruction is based on an average of five sites covering various periods of the last six millennia.

***Supplementary Table S1*│ Recorded data from the Akko core.** The pollen-derived ecosystems are displayed in percentages and presented on a linear age-scale.

***Supplementary Table S2*│ Data from the PCA-Axis1 and the reconstructed climate values for the Akko core.**

***Supplementary Table S3*│ Modern values for the reconstructed climate parameters.** The data the mean temperature of the coldest month (MTCO) and the mean temperature of the warmest month (MTWA)

***Supplementary Table S4*│ Mean temperature of the coldest month (MTCO) and mean temperature of the warmest month (MTWA): root-mean-square error (RMSE), bias and mean confidence interval (IC).**

Supplementary Table S5 **│ Reconstructed climate values for the Eastern Mediterranean.**

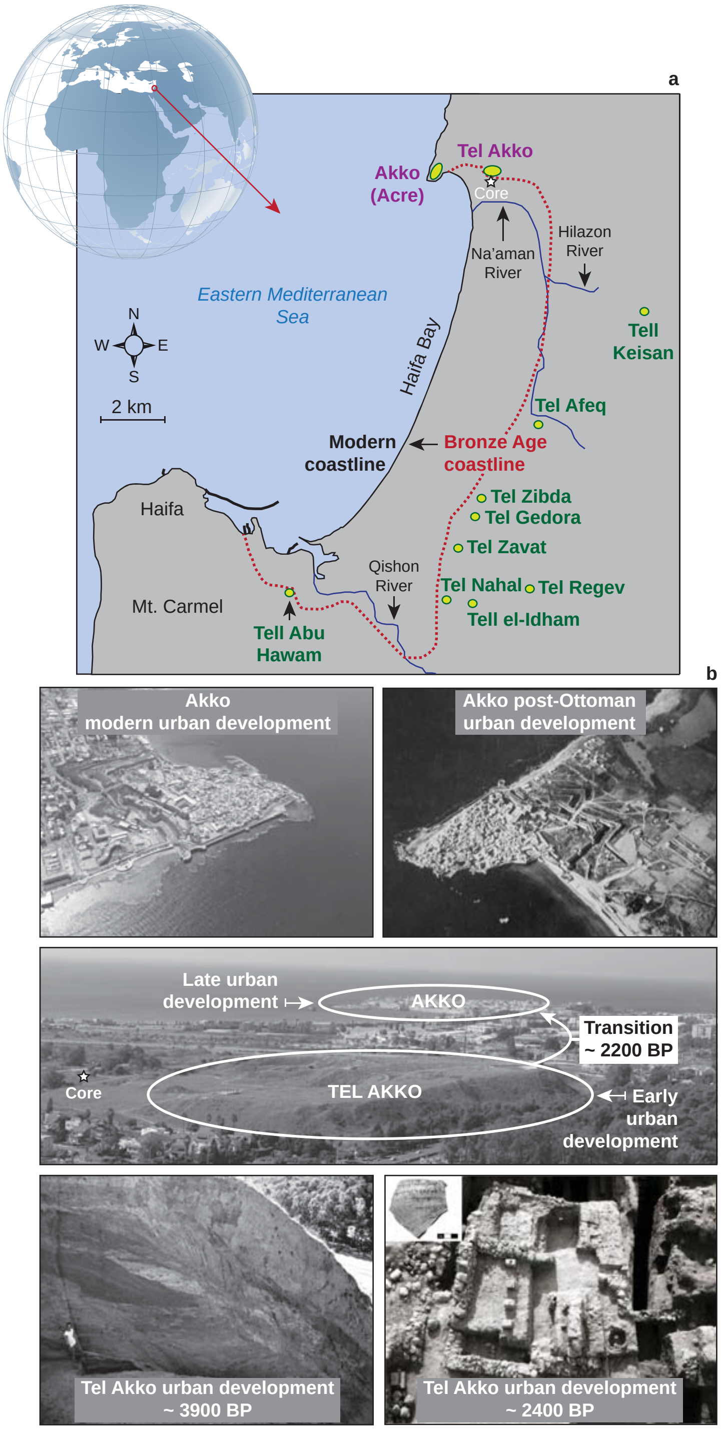
a
Tel Akko
Akko
(Acre)
Core
Hilazon
River
Na'aman
River
Eastern Mediterranean
Sea
N
W
E
S
2 km
Tell
Keisan
Haifa Bay
Tel Afeq
Modern
coastline
Bronze Age
coastline
Tel Zibda
Tel Gedora
Haifa
Tel Zavat
Qishon
River
Tel Nahal
Tel Regev
Tell el-Idham
Mt. Carmel
Tell Abu
Hawam
b
Akko
modern urban development
Akko post-Ottoman
urban development
Late urban
development
AKKO
Transition
~ 2200 BP
Core
TEL AKKO
Early
urban
development
Tel Akko urban development
~ 3900 BP
Tel Akko urban development
~ 2400 BP

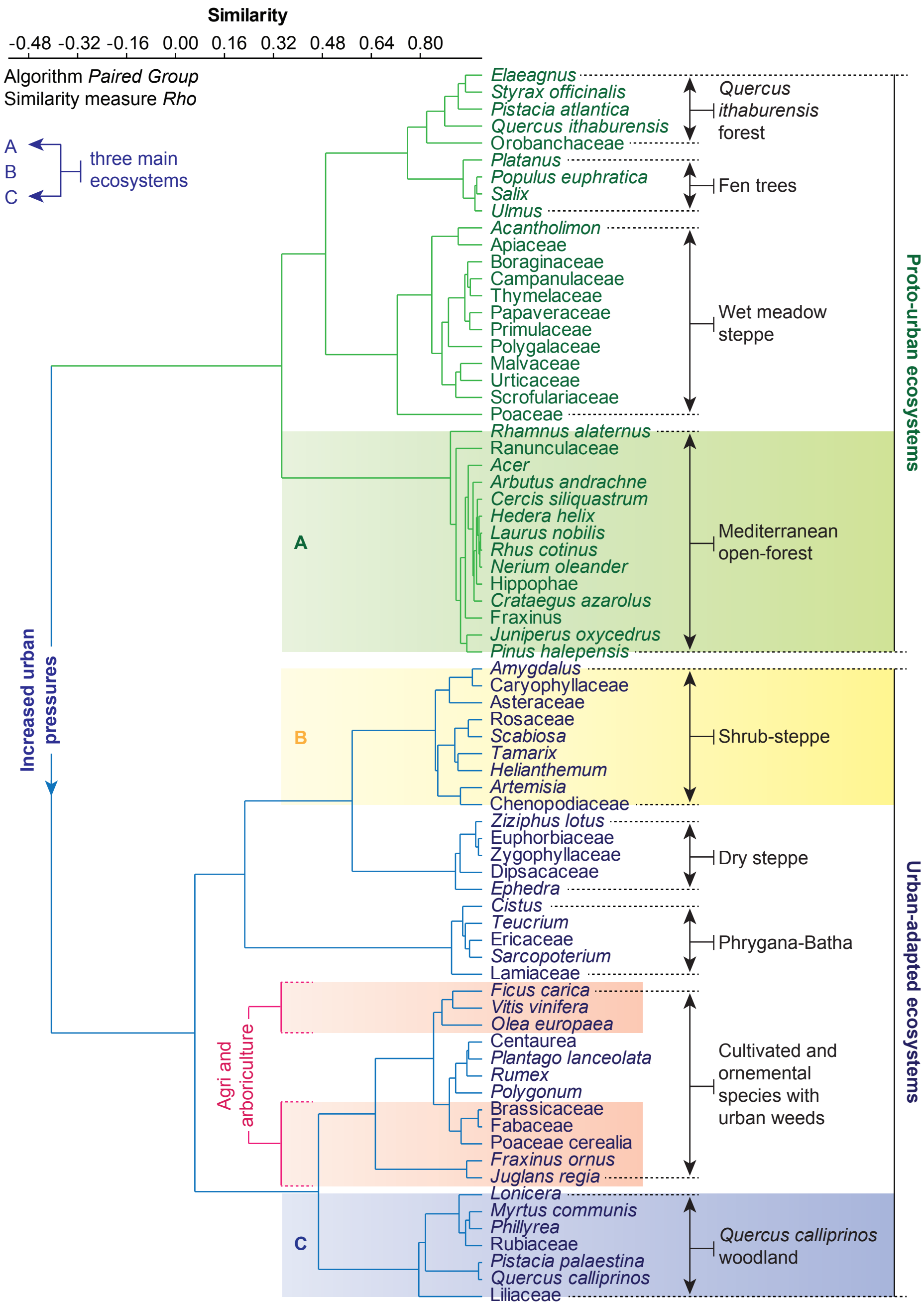
Similarity
-0.48 -0.32 -0.16 0.00 0.16 0.32 0.48 0.64 0.80
Algorithm Paired Group
Similarity measure Rho
A
B
C
three main ecosystems
Increased urban pressures
Elaeagnus
Styrax officinalis
Pistacia atlantica
Quercus ithaburensis
Orobanchaceae
Platanus
Populus euphratica
Salix
Ulmus
Acantholimon
Apiaceae
Boraginaceae
Campanulaceae
Thymelaceae
Papaveraceae
Primulaceae
Polygalaceae
Malvaceae
Urticaceae
Scrofulariaceae
Poaceae
Rhamnus alaternus
Ranunculaceae
Acer
Arbutus andrachne
Cercis siliquastrum
Hedera helix
Laurus nobilis
Rhus cotinus
Nerium oleander
Hippophae
Crataegus azarolus
Fraxinus
Juniperus oxycedrus
Pinus halepensis
Amygdalus
Caryophyllaceae
Asteraceae
Rosaceae
Scabiosa
Tamarix
Helianthemum
Artemisia
Chenopodiaceae
Ziziphus lotus
Euphorbiaceae
Zygophyllaceae
Dipsacaceae
Ephedra
Cistus
Teucrium
Ericaceae
Sarcopoterium
Lamiaceae
Ficus carica
Vitis vinifera
Olea europaea
Centaurea
Plantago lanceolata
Rumex
Polygonum
Brassicaceae
Fabaceae
Poaceae cerealia
Fraxinus ornus
Juglans regia
Lonicera
Myrtus communis
Phillyrea
Rubiaceae
Pistacia palaestina
Quercus calliprinos
Liliaceae
Agri and arboriculture
Quercus ithaburensis forest
Fen trees
Wet meadow steppe
Mediterranean open-forest
Shrub-steppe
Dry steppe
Phrygana-Batha
Cultivated and ornemental species with urban weeds
Quercus calliprinos woodland
Proto-urban ecosystems
Urban-adapted ecosystems

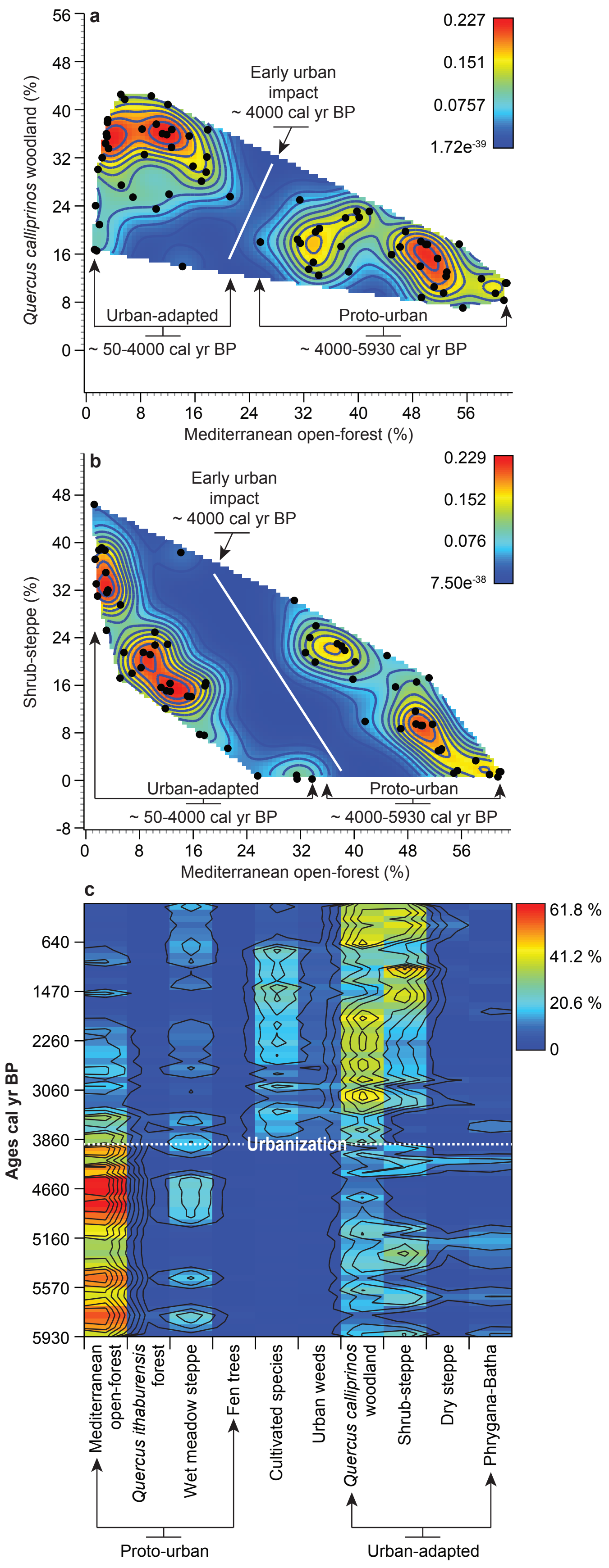
a
Quercus calliprinos woodland (%)
Mediterranean open-forest (%)
Early urban impact ~ 4000 cal yr BP
Urban-adapted
~ 50-4000 cal yr BP
Proto-urban
~ 4000-5930 cal yr BP
0.227
0.151
0.0757
1.72e-39
b
Shrub-steppe (%)
Mediterranean open-forest (%)
Early urban impact ~ 4000 cal yr BP
Urban-adapted
~ 50-4000 cal yr BP
Proto-urban
~ 4000-5930 cal yr BP
0.229
0.152
0.076
7.50e-38
c
Ages cal yr BP
Urbanization
61.8 %
41.2 %
20.6 %
0
Mediterranean open-forest
Quercus ithaburensis forest
Wet meadow steppe
Fen trees
Cultivated species
Urban weeds
Quercus calliprinos woodland
Shrub-steppe
Dry steppe
Phrygana-Batha
Proto-urban
Urban-adapted

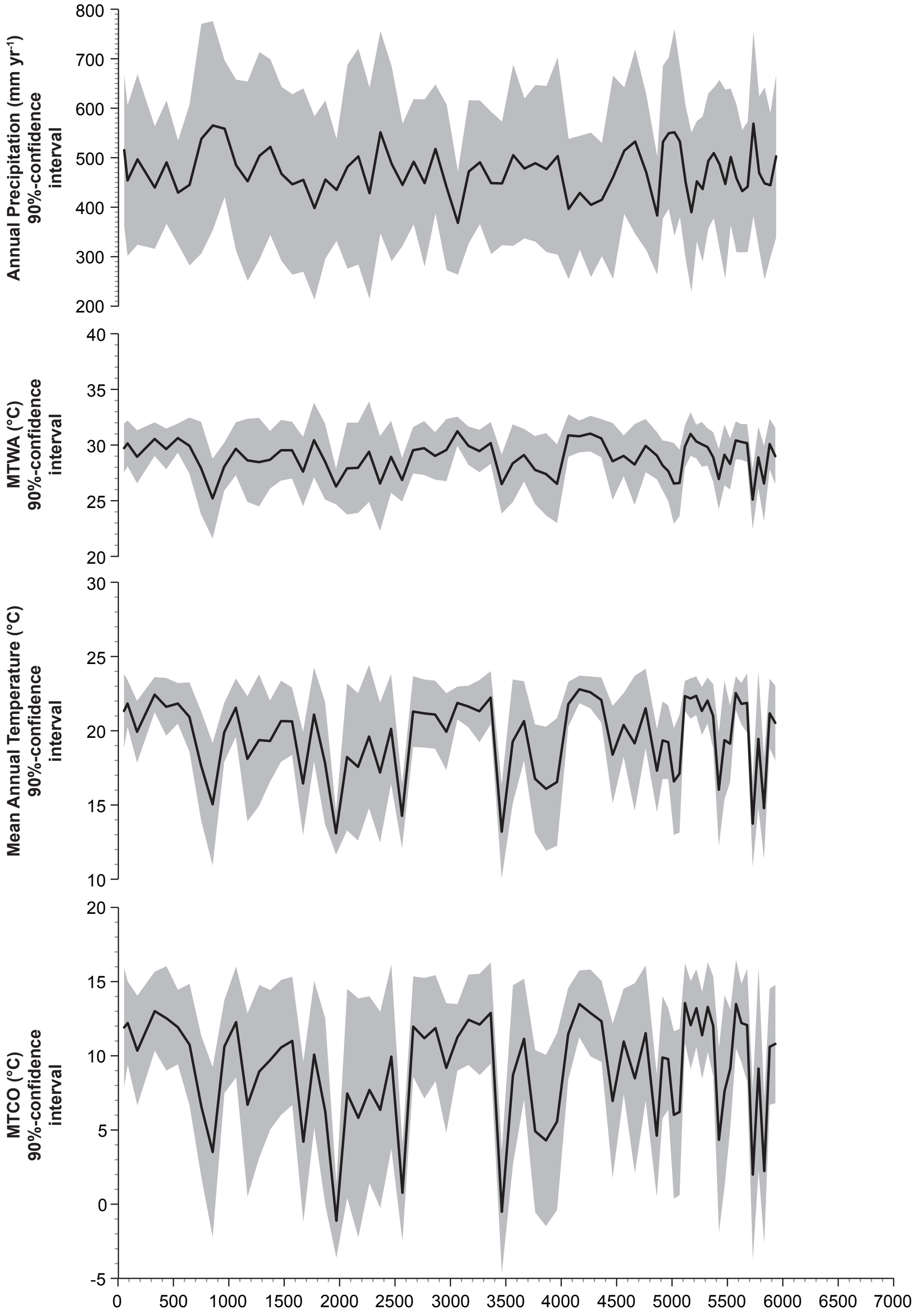

Annual Precipitation (mm yr-1) 90%-confidence interval
800
700
600
500
400
300
200
MTWA (°C) 90%-confidence interval
40
35
30
25
20
Mean Annual Temperature (°C) 90%-confidence interval
30
25
20
15
10
MTCO (°C) 90%-confidence interval
20
15
10
5
0
-5
0 500 1000 1500 2000 2500 3000 3500 4000 4500 5000 5500 6000 6500 7000
Ages cal yr BP

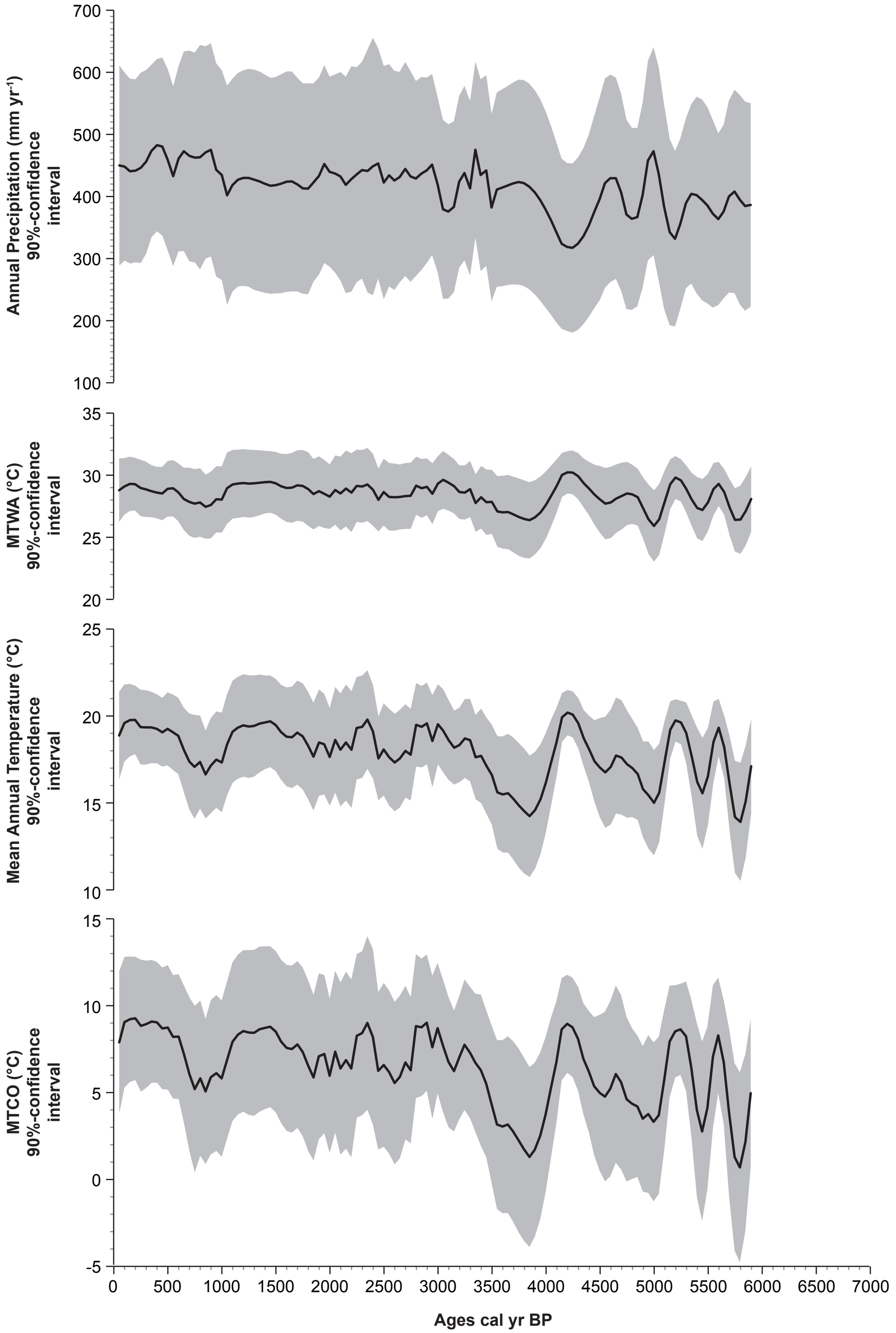
Annual Precipitation (mm yr-1) 90%-confidence interval
700
600
500
400
300
200
100
MTWA (°C) 90%-confidence interval
35
30
25
20
Mean Annual Temperature (°C) 90%-confidence interval
25
20
15
10
MTCO (°C) 90%-confidence interval
15
10
5
0
-5
0 500 1000 1500 2000 2500 3000 3500 4000 4500 5000 5500 6000 6500 7000
Ages cal yr BP

Supplementary Table S1

Med. open-forest = Mediterranean open-forest
Quercus itha. Forest = *Quercus ithaburensis* forest
Wet mead. steppe = Wet meadow steppe
Cult. species = Cultivated species
Quercus cal. Wood. = *Quercus calliprinos* woodland

Ages (cal yr BP)	Med. open-forest	*Quercus itha.* forest	Wet mead. steppe	Fen trees	Cult. species	Urban weeds	*Quercus cal.* wood.	Shrub-steppe	Dry steppe	Phrygana-Batha
50	3,2407	0,0000	14,8148	0,0000	6,7130	4,1667	33,3333	31,9444	0,2315	5,5556
82	2,8011	0,0000	10,0840	0,0000	5,6022	3,3613	34,1737	38,6555	5,3221	0,0000
170	3,0864	0,0000	11,1111	0,0000	5,5556	3,7037	37,6543	31,4815	7,4074	0,0000
327	2,2989	0,0000	8,4291	0,0000	4,9808	2,2989	31,8008	39,0805	11,1111	0,0000
432	2,9326	0,0000	10,5572	0,0000	5,2786	3,5191	35,7771	34,8974	7,0381	0,0000
537	3,1250	0,0000	11,2500	0,0000	6,2500	3,7500	38,1250	31,5625	5,9375	0,0000
641	5,0526	0,0000	14,3158	0,0000	10,9474	5,8947	42,3158	17,0526	1,6842	2,7368
746	6,8213	0,0000	13,6426	0,8186	24,1473	8,1855	25,2387	17,8718	0,0000	3,2742
851	10,2384	1,4025	10,9397	1,6830	21,0379	6,3114	23,2819	24,8247	0,0000	0,0000
956	12,1287	0,9901	11,8812	1,2376	17,5743	6,1881	25,7426	22,7723	1,2376	0,0000
1060	1,1952	0,0000	5,5777	0,0000	18,3267	6,3745	16,5339	46,4143	4,7809	0,7968
1165	1,8975	0,0000	5,6926	0,0000	19,1651	7,0209	20,6831	38,7097	3,6053	3,2258
1270	5,1188	0,0000	8,5923	0,0000	17,0018	6,0329	27,2395	29,4333	1,4625	5,1188
1369	1,4993	0,0000	10,7946	0,0000	26,8366	9,5952	16,3418	32,9835	1,9490	0,0000
1469	14,1058	0,0000	0,7557	0,0000	22,5441	8,0605	13,7280	38,2872	2,5189	0,0000
1568	1,3316	0,0000	1,0652	0,0000	23,8349	8,5220	23,8349	37,1505	4,2610	0,0000
1668	1,6787	0,0000	1,9185	0,0000	20,1439	7,1942	29,8561	30,9353	3,2374	5,0360
1767	3,0205	0,0000	3,0205	0,0000	18,1230	6,4725	35,2751	25,1348	2,3732	6,5804
1866	5,6309	0,3128	5,0052	0,3128	15,6413	5,8394	41,5016	21,3764	0,0000	4,3796
1966	8,1726	1,1938	8,8154	1,1019	18,0900	7,1625	36,5473	18,8246	0,0000	0,0000
2065	12,5114	2,7397	8,7671	2,4658	16,7123	6,7580	33,5160	16,1644	0,0000	0,0000
2164	11,1579	1,7895	10,1053	1,6842	17,1579	6,7368	35,7895	15,4737	0,0000	0,0000
2264	11,7991	1,9860	9,4626	0,7009	19,0421	9,3458	35,6308	11,9159	0,0000	0,0000
2363	15,0923	3,0402	7,1661	1,5201	15,6352	7,8176	35,3963	14,0065	0,0000	0,0000
2463	15,6627	2,8112	8,8353	1,8742	17,1352	9,1031	30,3882	13,9224	0,0000	0,0000
2562	12,5356	1,7094	6,5527	0,7123	15,9544	8,8319	36,4672	14,8148	0,8547	1,4245
2661	17,8295	3,3592	12,0155	2,3256	6,5891	4,7804	36,4341	16,2791	0,0000	0,0000
2761	17,7005	3,1770	9,9849	2,1180	12,1029	6,8079	31,9213	15,7337	0,1513	0,0000
2860	10,2632	1,3158	3,6842	0,0000	7,3684	6,8421	37,3684	22,6316	6,3158	4,2105
2960	16,9043	3,2587	5,2953	0,8147	19,1446	13,2383	27,9022	7,5356	3,0550	2,8513
3060	11,9639	1,1287	5,8691	0,0000	11,0609	8,3521	40,6321	14,8984	3,1603	2,9345
3160	9,5355	1,2225	3,4230	0,0000	6,8460	6,3570	42,0538	21,0269	5,6235	3,9120
3260	8,5153	1,0917	3,0568	0,0000	13,1004	9,1703	32,3144	21,3974	5,8952	5,4585
3360	17,6000	3,2000	5,2000	0,8000	18,8000	13,0000	29,4000	7,4000	1,8000	2,8000
3460	31,4394	6,8182	12,5000	3,7879	15,9091	10,9848	17,6136	0,0000	0,0000	0,0000
3560	25,5613	5,1813	14,1623	3,4542	13,6442	8,1174	17,7893	0,5181	0,0000	10,8808
3660	21,1132	4,0307	6,7179	1,3436	15,1631	9,0211	25,3359	5,1823	0,9597	10,9405
3760	33,6714	7,3022	13,3874	4,0568	12,3732	8,7221	19,4726	0,0000	0,0000	0,0000
3860	31,3559	6,3559	17,3729	4,2373	8,0508	6,3559	24,7881	0,6356	0,0000	0,0000
3960	49,2366	4,5802	15,6489	2,4809	1,3359	0,5725	8,5878	16,4122	0,0000	0,5725
4060	46,8966	1,6092	10,3448	0,0000	1,8391	1,1494	19,5402	8,5057	4,1379	5,9770
4160	34,1317	0,0000	1,7964	0,0000	2,0958	1,4970	12,2754	19,7605	15,2695	13,1737
4260	51,0934	0,1988	1,3917	0,0000	1,1928	0,7952	10,3380	17,0974	8,9463	8,9463
4360	46,1078	1,3972	8,9820	0,0000	1,3972	0,7984	16,9661	15,5689	3,5928	5,1896
4460	61,3757	4,7619	20,8113	2,1164	1,2346	0,8818	8,1129	0,3527	0,0000	0,0000
4560	60,1113	4,2672	21,8924	1,2987	1,2987	0,7421	9,2764	0,7421	0,0000	0,0000
4660	61,7886	3,2520	20,5285	0,0000	1,4228	0,6098	10,9756	1,2195	0,0000	0,0000
4760	54,7912	2,9484	20,8845	0,0000	1,7199	1,2285	17,4447	0,9828	0,0000	0,0000
4860	61,5854	3,2520	20,5285	0,0000	1,2195	1,0163	10,9756	1,2195	0,0000	0,0000
4911	58,0574	5,0773	18,3223	0,6623	1,5453	0,4415	11,6998	3,0905	0,0000	0,6623
4962	52,9801	5,0773	18,3223	1,5453	1,7660	0,8830	12,8035	5,0773	0,0000	1,1038
5013	49,1647	3,5800	13,3652	0,0000	1,4320	0,9547	17,8998	9,3079	0,4773	3,5800
5064	40,0922	0,9217	3,6866	0,0000	1,6129	0,9217	21,8894	19,8157	2,7650	8,2949
5115	41,5789	0,7895	2,3684	0,0000	1,8421	1,8421	22,8947	9,7368	6,3158	12,6316
5166	32,6861	0,0000	1,9417	0,0000	1,9417	1,2945	13,2686	21,3592	13,2686	14,2395
5217	33,3333	0,2778	1,9444	0,0000	2,2222	0,8333	14,4444	23,8889	10,5556	12,5000
5268	31,0273	0,6289	1,8868	0,0000	1,2579	0,8386	18,2390	30,1887	5,8700	10,0629
5319	34,2380	0,8351	3,3403	0,0000	1,4614	1,0438	20,0418	25,8873	4,8017	8,3507
5370	38,0734	0,9174	3,6697	0,0000	1,3761	0,9174	21,7890	22,4771	2,5229	8,2569
5420	50,1160	3,4803	12,9930	0,0000	1,3921	1,3921	17,4014	9,0487	0,4640	3,4803
5471	52,8662	5,3079	18,0467	1,9108	1,4862	1,0616	12,1019	4,8832	0,0000	1,6985
5522	49,8840	3,4803	12,9930	0,0000	1,8561	1,1601	17,4014	9,0487	0,4640	3,4803
5573	39,7590	0,9639	2,8916	0,0000	1,6867	0,9639	22,8916	16,8675	5,3012	8,6747
5624	38,5514	0,9346	2,8037	0,0000	1,6355	0,2336	12,8505	21,7290	9,8131	11,4486
5675	44,8430	1,7937	4,4843	0,0000	1,1211	0,4484	15,6951	20,8520	2,9148	7,8475
5726	51,6129	4,3011	11,3978	1,2903	1,5054	1,0753	15,0538	9,2473	0,8602	3,6559
5777	55,3320	7,6459	21,9316	3,0181	1,2072	1,0060	6,8410	1,4085	0,0000	1,0060
5828	52,4946	5,8568	18,6551	2,1692	1,5184	1,9523	9,3275	4,7722	0,0000	3,0369
5879	49,0654	3,5047	12,3832	1,1682	1,4019	1,1682	13,7850	11,4486	0,0000	6,0748
5930	37,4220	2,4948	4,5738	0,2079	1,2474	1,0395	17,0478	22,8690	1,6632	11,4345

Supplementary Table S2

Annual P = annual precipitation
MTCO = mean temperature of the coldest month
Annual T = mean annual temperature
MTWA = mean temperature of the warmest month

Dates (cal yr BP)	PCA-Axis 1 (scores)	Annual P (mm yr-1)	MTCO (°C)	Annual T (°C)	MTWA (°C)	Annual P (90% conf. int.)	MTCO (90% conf. int.)	Annual T (90% conf. int.)	MTWA (90% conf. int.)
50	-28,312	515,24	11,90	21,33	29,71	152,81	4,00	2,48	2,20
82	-32,477	454,01	12,20	21,83	30,13	152,19	2,80	1,57	2,06
170	-30,625	496,52	10,35	19,93	28,93	171,99	3,70	2,08	2,38
327	-32,681	439,57	13,00	22,43	30,53	123,56	2,66	1,18	1,51
432	-31,477	490,83	12,52	21,61	29,64	124,58	3,51	1,94	1,84
537	-30,713	429,63	11,93	21,83	30,62	104,75	2,51	1,37	1,31
641	-24,353	445,21	10,74	20,92	29,93	162,89	4,10	2,33	2,52
746	-17,345	538,46	6,59	17,66	27,92	232,28	4,76	3,66	4,16
851	-16,784	565,00	3,52	15,04	25,19	211,03	5,72	4,12	3,59
956	-15,193	558,76	10,63	19,88	28,04	138,15	3,14	1,99	2,15
1060	-31,331	484,62	12,27	21,54	29,66	172,80	3,73	1,96	2,37
1165	-29,101	452,57	6,69	18,11	28,62	201,44	6,18	4,22	3,74
1270	-24,57	503,72	8,95	19,37	28,45	209,63	5,81	4,43	3,97
1369	-25,108	521,71	9,73	19,30	28,67	177,26	4,69	2,73	2,59
1469	-16,942	467,13	10,56	20,64	29,53	176,01	4,55	2,73	2,72
1568	-30,779	446,16	11,00	20,63	29,53	182,38	4,32	2,27	2,54
1668	-29,901	455,06	4,21	16,45	27,61	185,52	5,40	3,48	3,10
1767	-28,191	398,47	10,09	21,09	30,44	185,41	4,96	3,17	3,36
1866	-26,357	455,79	6,23	17,82	28,50	159,65	6,28	4,12	3,42
1966	-21,075	435,21	-1,11	13,11	26,26	102,99	2,49	1,45	1,60
2065	-15,254	481,86	7,47	18,23	27,88	205,86	7,03	4,95	4,13
2164	-16,8	502,49	5,82	17,56	27,94	218,48	8,04	4,96	4,06
2264	-14,934	428,02	7,71	19,61	29,38	213,60	6,30	4,83	4,51
2363	-13,1	551,67	6,36	17,18	26,54	204,68	6,60	4,71	4,27
2463	-10,662	487,81	9,94	20,12	28,92	197,03	6,21	3,69	3,23
2562	-16,105	445,34	0,77	14,26	26,86	123,31	3,26	2,15	1,96
2661	-11,33	492,12	11,97	21,28	29,52	126,50	3,38	2,38	2,07
2761	-9,9816	448,85	11,19	21,16	29,72	169,09	4,06	2,29	2,41
2860	-21,86	517,94	11,86	21,08	29,01	130,28	3,57	2,29	2,13
2960	-6,8282	439,94	9,18	19,93	29,54	167,36	4,37	2,59	2,79
3060	-18,224	368,24	11,24	21,87	31,22	104,38	2,24	1,10	1,31
3160	-23,486	471,89	12,42	21,63	29,93	144,42	3,04	1,40	1,72
3260	-21,192	490,82	12,11	21,31	29,45	125,02	3,41	2,09	1,91
3360	-6,6732	449,00	12,88	22,22	30,17	143,85	3,41	1,80	1,89
3460	13,101	448,15	-0,51	13,21	26,49	125,08	4,14	3,16	2,66
3560	8,2534	505,24	8,71	19,27	28,35	183,32	6,04	4,18	3,50
3660	-0,98571	478,35	11,14	20,65	29,08	141,48	4,05	2,67	2,36
3760	14,504	488,67	4,92	16,77	27,75	157,95	5,47	3,66	3,05
3860	10,966	477,08	4,30	16,09	27,38	167,83	5,76	4,18	3,70
3960	25,25	503,61	5,57	16,55	26,51	199,38	5,96	4,30	3,54
4060	21,608	396,61	11,46	21,78	30,85	141,60	3,10	1,51	1,91
4160	7,5423	428,97	13,49	22,78	30,77	115,02	2,25	0,94	1,43
4260	23,639	404,87	12,89	22,58	31,01	145,65	2,92	1,07	1,60
4360	18,933	415,27	12,32	22,06	30,60	114,31	2,73	1,52	1,73
4460	42,479	460,79	6,97	18,39	28,52	205,24	5,19	3,28	3,45
4560	40,969	514,20	10,97	20,39	29,01	127,90	3,55	2,14	1,85
4660	41,365	532,56	8,49	19,15	28,25	186,63	6,39	4,54	3,63
4760	33,359	470,00	11,53	21,50	29,93	157,26	4,55	2,67	2,38
4860	41,193	383,33	4,62	17,31	29,03	119,65	4,12	2,79	2,13
4911	37,089	531,98	9,89	19,35	28,16	154,29	4,11	2,61	2,23
4962	31,684	549,62	9,79	19,23	27,64	153,18	3,43	2,48	2,69
5013	24,376	551,53	6,02	16,58	26,54	208,95	5,65	3,60	3,61
5064	9,9154	532,52	6,21	17,10	26,58	152,38	5,59	3,96	2,99
5115	14,465	449,95	13,55	22,33	29,85	154,36	2,70	1,01	1,91
5166	5,4873	389,74	12,06	22,17	30,98	161,52	2,96	1,36	1,96
5217	4,7404	452,17	13,21	22,34	30,31	121,98	2,62	1,30	1,51
5268	-0,78322	436,47	11,38	21,32	30,05	146,56	2,93	1,62	2,01
5319	3,159	493,58	13,28	22,02	29,81	136,43	3,03	1,54	1,69
5370	7,2332	509,66	12,00	20,98	28,91	131,64	3,35	2,17	2,23
5420	25,391	486,55	4,33	16,03	26,92	170,35	6,22	3,81	2,70
5471	31,898	446,87	7,65	19,36	29,11	190,86	6,44	4,00	2,73
5522	25,195	501,81	9,16	19,12	28,29	137,82	3,96	2,56	2,30
5573	10,23	458,44	13,49	22,53	30,41	149,08	2,98	1,14	1,70
5624	10,675	432,64	12,20	21,80	30,26	123,63	2,64	1,40	1,60
5675	15,793	441,30	12,06	21,87	30,16	131,66	3,81	2,00	1,70
5726	27,226	568,88	2,00	13,75	25,10	186,89	5,73	2,93	2,69
5777	37,761	468,93	9,14	19,45	28,89	154,86	6,86	4,61	2,76
5828	32,724	447,89	2,24	14,78	26,54	194,29	4,88	3,41	3,38
5879	24,836	444,56	10,60	21,16	30,08	145,67	3,90	2,33	2,28
5930	8,4767	502,76	10,80	20,52	28,99	164,13	3,99	2,51	2,52

Supplementary Table S3

	Annual Precipitation (mm yr^{-1})	Mean annual temperature (°C)	MTCO (°C)	MTWA (°C)
**Akko**	451.353	21.405	11.429	30.174
**Eastern Mediterranean**	449.606	18.456	7.292	28.667

Supplementary Table S4

Reconstructed variable	RMSE	Bias	Corrected RMSE	Mean IC (90%) EasternMediterranean	Mean IC (90%) Akko
**MTCO °C**	9.7	-8.8	4.2	4.3	4.3
**MTWA °C**	1.8	0.7	1.7	2.5	2.6

Supplementary Table S5

Annual P = annual precipitation
MTCO = mean temperature of the coldest month
Annual T = mean annual temperature
MTWA = mean temperature of the warmest month

Ages (cal yr BP)	Annual P (mm yr-1)	MTCO (°C)	Annual T (°C)	MTWA (°C)	Annual P (90% conf. int.)	MTCO (90% conf. int.)	Annual T (90% conf. int.)	MTWA (90% conf. int.)
50	449,96	7,89	18,87	28,79	161,27	4,10	2,53	2,56
100	448,09	9,05	19,59	29,09	151,24	3,75	2,22	2,28
150	440,74	9,22	19,76	29,30	148,76	3,60	2,09	2,18
200	441,37	9,26	19,77	29,27	147,45	3,54	1,97	2,13
250	446,09	8,84	19,36	28,97	153,27	3,79	2,15	2,26
300	455,82	8,93	19,34	28,85	147,73	3,66	2,14	2,23
350	473,16	9,09	19,34	28,71	138,95	3,53	2,05	2,10
400	482,60	9,03	19,26	28,60	138,54	3,47	1,98	2,08
450	480,28	8,69	19,06	28,53	143,23	3,49	1,98	2,15
500	459,35	8,74	19,26	28,89	145,90	3,58	2,10	2,24
550	432,59	8,20	19,05	28,95	144,70	3,56	2,17	2,27
600	460,61	8,22	18,85	28,61	148,99	3,59	2,20	2,34
650	472,91	7,21	18,05	28,09	160,62	3,94	2,43	2,52
700	465,14	6,05	17,38	27,85	169,64	4,42	2,76	2,74
750	462,69	5,20	17,07	27,71	168,93	4,79	2,99	2,77
800	463,03	5,82	17,35	27,80	180,50	4,46	2,68	2,75
850	470,94	5,06	16,63	27,45	170,74	4,17	2,52	2,58
900	474,96	5,88	17,14	27,59	171,67	4,44	2,74	2,68
950	442,69	6,12	17,49	28,05	171,68	4,48	2,77	2,66
1000	434,29	5,81	17,33	28,04	168,90	4,50	2,86	2,68
1050	401,86	6,90	18,35	28,95	176,65	4,52	2,91	2,87
1100	418,31	7,92	19,09	29,25	171,42	4,56	2,94	2,77
1150	427,02	8,34	19,32	29,31	171,89	4,59	2,93	2,74
1200	429,59	8,54	19,45	29,36	173,43	4,65	2,95	2,74
1250	430,03	8,45	19,39	29,32	175,57	4,73	2,95	2,74
1300	426,83	8,44	19,42	29,33	177,09	4,77	2,91	2,70
1350	424,09	8,64	19,56	29,38	177,08	4,75	2,81	2,62
1400	420,27	8,72	19,63	29,43	175,59	4,68	2,69	2,53
1450	417,11	8,79	19,69	29,46	173,86	4,62	2,63	2,48
1500	417,93	8,50	19,47	29,34	174,23	4,63	2,65	2,52
1550	420,36	7,94	19,07	29,11	176,13	4,70	2,73	2,62
1600	423,74	7,57	18,80	28,96	177,66	4,77	2,84	2,73
1650	424,07	7,50	18,78	28,98	176,96	4,81	2,92	2,81
1700	419,43	7,76	19,04	29,17	172,16	4,79	2,98	2,84
1750	412,78	7,34	18,82	29,14	169,14	4,85	3,05	2,89
1800	412,33	6,53	18,28	28,87	169,62	4,90	3,10	2,96
1850	422,31	5,86	17,66	28,47	159,76	4,76	3,09	2,84
1900	432,91	7,09	18,47	28,71	158,51	4,77	3,06	2,80
1950	452,14	7,21	18,38	28,49	159,40	4,55	2,90	2,74
2000	439,83	5,97	17,64	28,26	152,99	4,44	2,81	2,59
2050	436,81	7,35	18,62	28,80	159,73	4,65	3,04	2,79
2100	432,31	6,38	18,05	28,53	167,77	4,95	3,25	2,97
2150	418,55	6,86	18,47	28,92	174,26	5,09	3,34	3,06
2200	428,13	6,39	18,06	28,59	181,61	5,11	3,36	3,18
2250	435,30	8,27	19,30	29,14	173,07	4,70	2,94	2,90
2300	442,31	8,41	19,38	29,09	174,55	4,71	2,94	2,93
2350	441,09	9,00	19,79	29,25	195,72	4,99	2,84	2,95
2400	448,27	8,19	19,10	28,82	206,92	5,04	2,69	2,90
2450	453,26	6,25	17,56	28,00	185,49	4,52	2,41	2,54
2500	422,24	6,59	18,07	28,63	187,64	4,71	2,65	2,70
2550	434,02	6,18	17,62	28,25	178,79	4,69	2,79	2,72
2600	425,71	5,55	17,32	28,23	176,36	4,67	2,80	2,70
2650	431,28	5,89	17,55	28,24	169,68	4,68	2,81	2,62
2700	444,28	6,73	17,99	28,32	172,18	4,41	2,69	2,65
2750	432,21	6,28	17,77	28,34	169,62	4,20	2,64	2,67
2800	428,92	8,82	19,49	29,15	156,75	4,14	2,41	2,33
2850	437,01	8,75	19,38	28,94	155,22	3,95	2,34	2,37
2900	442,16	9,01	19,57	29,06	149,06	3,92	2,34	2,30
2950	451,35	7,60	18,55	28,50	145,60	4,12	2,64	2,45
3000	418,93	8,70	19,53	29,32	140,72	3,75	2,30	2,22
3050	379,15	7,66	19,15	29,61	143,94	3,81	2,39	2,32
3100	375,70	6,76	18,61	29,38	140,31	3,75	2,29	2,24
3150	383,27	6,24	18,18	29,10	137,60	3,48	2,13	2,18
3200	423,54	7,01	18,33	28,63	138,63	3,54	2,15	2,16
3250	437,53	7,75	18,70	28,60	140,46	3,75	2,27	2,19
3300	412,94	7,29	18,60	28,88	140,49	3,78	2,37	2,25
3350	475,31	6,71	17,63	27,76	141,63	3,97	2,43	2,31
3400	434,31	6,28	17,71	28,22	154,33	4,35	2,72	2,52
3450	441,79	5,51	17,14	27,82	153,56	4,22	2,76	2,63
3500	382,20	4,30	16,61	27,85	150,71	4,54	2,88	2,57
3550	411,03	3,16	15,60	27,08	156,49	4,85	3,24	2,85
3600	414,29	3,04	15,49	27,01	158,50	4,98	3,34	2,93
3650	417,69	3,16	15,55	27,02	160,05	5,08	3,42	2,99
3700	420,91	2,76	15,24	26,83	162,42	5,19	3,51	3,05
3750	423,11	2,24	14,85	26,62	165,16	5,29	3,59	3,11
3800	421,49	1,75	14,52	26,46	165,77	5,27	3,58	3,11
3850	415,82	1,29	14,23	26,37	165,56	5,17	3,50	3,07
3900	406,56	1,71	14,58	26,61	164,48	5,01	3,36	2,99
3950	394,40	2,53	15,22	27,01	162,39	4,79	3,16	2,88
4000	379,45	3,73	16,15	27,59	158,44	4,47	2,87	2,70
4050	361,45	5,32	17,37	28,35	152,17	4,01	2,45	2,44
4100	342,26	6,75	18,49	29,11	145,17	3,50	1,99	2,15
4150	323,90	8,64	19,92	30,02	136,83	2,94	1,41	1,78
4200	318,43	8,95	20,19	30,23	135,32	2,82	1,30	1,71
4250	317,09	8,75	20,08	30,21	136,28	2,85	1,33	1,76
4300	323,81	8,09	19,57	29,90	138,72	2,99	1,45	1,86
4350	336,01	6,95	18,70	29,35	141,78	3,18	1,63	1,98
4400	352,80	6,20	18,09	28,92	146,51	3,48	1,91	2,14
4450	374,22	5,36	17,39	28,42	154,72	3,97	2,36	2,41
4500	395,86	4,98	17,03	28,06	163,19	4,49	2,84	2,69
4550	421,27	4,77	16,76	27,71	168,55	4,88	3,19	2,87
4600	429,13	5,22	17,07	27,79	167,11	5,04	3,31	2,87
4650	429,49	6,06	17,72	28,10	162,10	5,10	3,34	2,76
4700	406,65	5,59	17,61	28,31	158,53	5,02	3,29	2,69
4750	371,15	4,61	17,23	28,52	152,48	4,72	3,08	2,54
4800	363,93	4,37	17,00	28,46	146,52	4,34	2,82	2,38
4850	366,78	4,20	16,66	28,22	143,47	4,06	2,64	2,29
4900	402,27	3,49	15,78	27,36	149,20	4,18	2,76	2,46
4950	457,90	3,76	15,44	26,45	160,79	4,54	3,08	2,79
5000	472,74	3,31	15,01	25,91	167,18	4,57	3,03	2,87
5050	435,56	3,70	15,58	26,45	171,94	4,52	2,83	2,85
5100	383,08	5,78	17,40	27,87	164,61	4,02	2,35	2,53
5150	342,34	7,94	19,21	29,27	149,85	3,21	1,63	2,00
5200	331,92	8,52	19,74	29,81	141,45	2,65	1,22	1,72
5250	356,20	8,63	19,63	29,58	137,17	2,62	1,25	1,70
5300	389,21	8,24	19,03	28,89	136,78	3,14	1,75	1,90
5350	404,26	6,43	17,68	28,04	144,30	3,91	2,34	2,14
5400	402,33	4,00	16,20	27,36	158,41	4,99	3,10	2,46
5450	394,16	2,77	15,55	27,19	161,66	5,16	3,19	2,50
5500	385,64	4,12	16,53	27,80	158,15	4,67	2,79	2,32
5550	372,20	7,06	18,51	28,87	150,99	4,12	2,32	2,10
5600	363,37	8,29	19,33	29,30	138,29	3,32	1,65	1,78
5650	375,78	6,74	18,21	28,64	139,73	3,57	1,79	1,84
5700	399,93	3,87	16,06	27,38	154,61	4,82	2,74	2,26
5750	408,05	1,30	14,19	26,40	163,61	5,39	3,23	2,55
5800	394,83	0,70	13,91	26,41	168,54	5,46	3,40	2,74
5850	384,28	2,16	15,08	27,10	168,45	5,08	3,23	2,76
5900	386,50	4,97	17,11	28,07	163,55	4,26	2,72	2,62
